# Remarks on Cerebro-Spinal Meningitis, as It Prevailed in and near San Augustine, Texas, in the Spring of 1852

**Published:** 1853-06

**Authors:** Isaiah J. Roberts


### WESTERN JOURNAL

OF

MEDICINE AND SURGERY.

JUNE, 1853.

Art. I.—*Remarks on Cerebro-Spinal Meningitis*, as it prevailed in and near San Augustine, Texas, in the spring of 1852. Submitted to the Trustees and Medical Faculty of the University of Louisville, February 1st, 1853, for the degree of *Doctor of Medicine*. By Isaiah J. Roberts, M. D.

This disease was confined to a comparatively small district of country, not extending over an area of more than twelve or fifteen square miles. It first made its appearance about the middle of March, and prevailed with great malignity until the last of May.

The previous winter had been noted for the great abundance of rain, as well as the extreme coldness of the weather for the climate. In the early part of the spring the changes were very sudden and extremely severe, the thermometer frequently falling twenty or thirty degrees in a few hours, and not unfrequently thirty or forty degrees in the course of twenty-four hours; changes very unusual even in a climate where sudden changes of temperature are not unfrequent.

For several weeks previous to the appearance of the disease, there had been a prevalence of diarrhœa, which seemed to be epidemic in its character, very difficult to control by medicine, but not fatal. Whether there was any connection between the two diseases or not is not easy to determine, but very suddenly the complaint under consideration made its appearance, several cases occurring about the same time in the neighborhood, and the diarrhœa as suddenly subsided.

The mode of attack was very sudden in every case, the person appearing as well as usual, probably pursuing his daily avocation, until the very onset of the disease, when he would be stricken down, and was frequently almost senseless and motionless from the very commencement of the attack, requiring assistance, in many cases, to get to his bed or house, though he might have been but a short distance off at the time of the access of the disease; the patient walking with a peculiar staggering gait, as though he were under the influence of alcohol or some narcotic poison, and frequently being unable to walk at all without assistance.

The disease seemed to be confined to the most healthy, robust young persons, there having been but two or three instances of persons over eighteen years of age attacked by it, and generally they were under fifteen; and what is more extraordinary, there was not a single instance of the disease appearing in a female, though seemingly exposed to the same causes as the males, so far as epidemic influences were concerned; neither was there an instance of the disease occurring in negroes, who were probably more exposed to the vicissitudes of the weather than the whites.

The symptoms were very similar in every case; the commencement of the disease was with a chill, or state of collapse, which generally lasted from six to twelve hours, and in some cases as long as twenty-four hours, during the continuance of which the patient was seized with the most intense pain in the head and upper part of the spinal column. In the midst of the chill there was generally some moisture on the surface of the body, especially the forehead, with a small feeble pulse and difficult breathing, a feeling of oppression in the chest, nausea and sometimes vomiting ; some of the symptoms resembling very nearly those which occur in the common congestive fever, which frequently prevails in this section of the country. The patient generally complained of ringing in the ears, imperfect vision, objects appearing double or only half an object being seen, or in other cases every thing seeming surrounded by a mist ; pupils of the eyes dilated ; more or less deafness, and disinclination to speak or move ; frequent exclamations of pain ; some stupor, and when aroused the patient soon relapsing into the stupor again.

The chills were followed by febrile excitement, not generally very high, with difficulty of moving the extremities; soreness all over the body ; pulse full and hard, from ninety to one hundred per minute, with strong determination to the head ; throbbing of the carotid and temporal arteries, with increase of the pain in the head and upper part of the spinal column ; attended by vertigo, delirium, and great restlessness ; the tongue but little coated, but constant complaint of thirst, the patient not being able to be moved in bed or handled rudely, without feeling great pain. If the nausea and vomiting did not occur during the chill, they commenced in almost all cases within the first twenty-four or forty-eight hours after the development of fever, attended by soreness on pressure over the epigastrium ; prehension difficult, it being with the utmost difficulty, in many cases, that the patient could take a glass and drink water unassisted.

In some cases there were involuntary twitchings of the muscles, when the patient attempted to move or seize hold of anything, as if under the influence of strychnia; retraction of the head, owing to the contraction and rigidity of the muscles of the neck. To such an extent was this rigidity of the muscles of the neck carried, in some cases, that by taking hold of the pillow by the ends and raising it up, the whole body of the patient might be raised without bending the neck. Great difficulty, and in some cases entire inability, to turn the head from side to side; soon after the development of fever, the pupils of the eyes, instead of being dilated as during the chill, became contracted and irritable to the light, attended with ringing in the ears.

As is usual on the appearance of a new epidemic, or form of disease, there was a difference of opinion with the physicians as to the cause and nature of the complaint, which led to different modes of treatment. Some believing the disease to be a form of congestive fever, malignant in its character and principally affecting the brain, treated it as such by bleeding, in some instances followed by tonics and stimulants, such as large doses of quinine, camphor, opium, cayenne pepper, brandy, etc., with stimulating applications externally applied. In some cases the stimulant course of treatment was commenced without the bleeding, but under this treatment the patients survived but a short time, violent reaction taking place which soon produced such results as destroyed life.

That the disease was not of the nature of congestive fever appears sufficiently evident from its course, there having been no instance of the chill returning after entire reaction had taken place, with development of fever, as would invariably have been the case in congestive fever, unless arrested by the appropriate treatment.—That there was active congestion of the brain, in the early stage of the disease there can be no doubt, but not such as belongs to congestive fever.

The disease did not appear to be much influenced by treatment, the result being the same—the death of the patient. The most marked difference was that the patients who were actively depleted in the early stage lived longer than those that were not, the cases in which depletion was not resorted to frequently terminating fatally in from four to eight days, while those patients who were actively depleted in the early stage lived generally twelve, fifteen, or twenty days, but eventually died. So fatal did the disease prove, that there was but a single instance of recovery out of twelve or fifteen cases.

The most rational treatment, and that which appeared to afford most relief, was the application of warmth externally, mustard plasters to the extremities and spine, warm pediluvia of mustard, frictions, etc., and after sufficient reaction had taken place to abstract blood, both generally and locally, from the temples and spine. So soon did reaction take place, after the first bleeding, in many instances, that a repetition of the process was called for in a few hours, and in such cases cold water appeared to afford relief; it was kept constantly applied, after the development of fever, either by pouring it in a small stream upon the head, or by using cloths dipped in cold water and applied frequently to the forehead.

In most cases venesection had to be frequently repeated, reaction soon taking place after each bleeding, the pulse becoming full and hard, with increase of the pain in the head and spine, and also increased restlessness and delirium. In the only case of recovery from the disease, the patient was bled two hundred ounces in the first two or three days; he was a young man, of full habit, seventeen years of age.

A solution of tartar emetic was exhibited, when the stomach would tolerate it, and in such doses as could be borne without producing too much nausea and vomiting. In most of the cases there was more or less nausea and vomiting from the disease, so that the tartar emetic could not be given regularly. Calomel was generally given, either alone, or in combination with the compound extract of colocynth, and in such doses in the early stage of the disease as to produce free purging. The calomel was continued in most cases until it produced ptyalism, after which the bowels were regulated by compound extract of colocynth, castor oil or salts. The constitutional effects of mercury were easily induced in most cases, but the disease did not appear to be arrested or much affected by ptyalism.

After reducing the system by general and local depletion, blisters were applied to the back of the neck and along the spine, sometimes its entire length, and kept running by irritating dressings. The tongue was seldom much coated at any stage of the disease, but was generally dry, the patient complaining of constant thirst. The diet, in the early stage, consisted of the lightest farinaceous articles, as rice, arrow-root and tapioca, but later in the disease more nourishing diet was administered.

Opium and morphine were tried in a few cases, but without any good result; they rather increased the stupor without relieving the pain and restlessness.

During the early stage of the disease the pupils of the eyes remained contracted and irritable to the light, the head retracted, the muscles of the neck and spine rigid, with inability to turn the head from side to side; intense pain in the head, inclination to sleep a great deal, with some stupor; throbbing of the carotids and temporal arteries; some rigidity of the muscles of the extremities, with a seeming inability to control their movements; great complaint on being moved or turned in bed; no appetite; continual thirst; pulse hard and quick, from one hundred to one hundred and twenty per minute. But generally, if the patient survived the second week, the pain in the head, which had been incessant, subsided; the pupils of the eyes, instead of continuing contracted as they had been during the previous course of the disease, became dilated and insensible to the light; the patient grew more stupid, semi-comatose, complaining of nothing, and apparently unconscious of any thing that was transpiring around him; the pulse became more slow and oppressed, slower in some cases than in health, although it had been as high as one hundred and twenty or more per minute. In some cases the patient on being moved manifested a disposition to spasm; in some cases there was difficulty in urinating, but only occasionally involuntary discharges of urine or fæces.

From this stage the patient gradually declined, the rigidity of the muscles of the neck and extremities continuing or increasing, the patient becoming completely comatose, unable to swallow or speak, living ten or fifteen days in some instances; the pulse becoming more slow and feeble, and the patient dying with symptoms of effusion on the brain, which no doubt was the case; but there was no opportunity to verify the fact by a post mortem inspection.

